# Relowering of Serum Na for Osmotic Demyelinating Syndrome

**DOI:** 10.1155/2012/704639

**Published:** 2012-06-28

**Authors:** Hideomi Yamada, Koji Takano, Nobuhiro Ayuzawa, George Seki, Toshiro Fujita

**Affiliations:** Department of Nephrology and Endocrinology, Faculty of Medicine, University of Tokyo, 7-3-1 Hongo, Bunkyo-ku, Tokyo 113-8655, Japan

## Abstract

We report a case in whom slow correction of hyponatremia (5 mmol/day for 3 days) induced central pontine myelinolysis (CPM). After the diagnosis was confirmed by imaging, we started to relower serum Na that completely recovered the sign and symptoms of CPM. Rapid correction of serum sodium is known to be associated with CPM. However, it may occur even after slow correction of hyponatremia. Currently, there is no standard therapy for CPM other than supportive therapy. Other therapy includes sterioid, plasmaphresis and IVIG, but these therapies have not been shown to be particularly effective. The pathophysiology of CPM is related to a relative dehydration of the brain during the correction of hyponatremia, resulting in cell death and demyelination, therefore gentle rehydration with lowering serum sodium may not be an unreasonable therapy. The present case provides supportive evidence that reinduction of hyponatremia is effective in treating CPM if started immediately after the diagnosis is suggested. The present case tells us that severe chronic hyponatremia must be managed with extreme care especially in patients with chronic debilitating illness and that relowering serum Na is a treatment of choice when CPM is suggested.

In September 2009, a 61-year-old housewife in end-stage renal failure was admitted to our hospital for disturbed consciousness by ambulance. Two weeks before admission, she caught cold and suffered from severe nausea, vomiting, and diarrhea, and finally became somnolent. Physical examination revealed a lethargic elderly woman with slight pretibial oedema, blood pressure 132/82 mmHg and pulse 92 beats/min, sinus rhythm. She had no remarkable past medical history.

Blood and urine analysis revealed severe hyponatremia (109 mmol/L), serum creatinine of 415.9 *μ*mol/L, BUN of 20.39 mmol/L, serum osmolality of 238 mOsm/L and plasma ADH of 0.86 pg/mL together with inappropriately high urine osmolality (159 mOsm/kg) and urine sodium concentration (25 mmol/L).

Her lethargy was attributed to severe hyponatremia caused by excessive ADH secretion stimulated by emesis. She was started with continuous infusion of 3% sodium chloride at 25 mL per hour, which increased serum Na by 5 mmol/day for the next three days. She became clear and alert without any neurological symptoms on the 3 d of admission (Figure 1(a)). However, on the 4 d, she began to complain of increased thirst. From the 5 d to 7 d, she became unable to speak or walk and did not move her body on the bed. Neurological examination revealed disturbed consciousness (E1V1M4), spastic tetraparesis, dysarthria, dysphagia, persistent nystagmus, and impaired bulbar movement. Axial, T2-weighted MR image of the pons revealed three linear hyperintense lesions in the center (Figure 1(b)). Auditory brainstem response revealed symmetrical delay of the I–III response. These results confirmed the diagnosis of central pontine myelinolysis (CPM). Her serum Na was 124 mmol/L. We decided to relower serum Na with a hope that it may recover some of the reversible damages that osmotic stress had caused [1]. When her serum Na was reduced to 117 mmol/L, she became remarkably better, started to speak, and moved her extremities upon request. Serum Na was set at low level (120 mmol/L) for additional 10 days. On 14 d, she became clear and alert, sat on bed, and spoke almost normally. On 21 d she walked by herself. The serum Na was gradually increased to 137 mmol/L during the next 2 weeks. On 35 d, she was discharged on foot without any residual signs and symptoms.

Osmotic demyelination syndrome, demonstrating as CPM, is a devastating neurologic disorder that often develops after rapid correction of chronic hyponatremia. Slow correction of serum Na (less than 12 mmol/L/day) is recommended to prevent the emergence of this syndrome [2]. However, it may occur even after slow correction of hyponatremia. In our case, serum Na correction by 5 mmol/day for three days developed CPM. Two similar cases are reported in patients with chronic renal failure [3]. In our case, relowering of serum Na by 7 mmol effectively recovered the patient's condition and finally restored all the neurological deficits. Immediate start of relowering may have assisted the recovery course. CPM is suggested to develop by the intracellular dehydration of oligodendrocytes in the pons and other susceptible area by hyperosmotic stimulus, which induces oligodendrocyte apoptosis and demyelination [4]. Glucocorticoid treatment is suggested but the effectiveness is not satisfactory. A case report suggested that reinduction of hyponatremia is effective in restoring the neurological deficits [1]. In experimental osmotic demyelination model, reinduction of hyponatremia resulted in significant decrease in mortality, whereas dexamethasone treatment did not alter mortality [5]. The present case provides supportive evidence that reinduction of hyponatremia is effective in treating CPM if started immediately after the diagnosis is suggested. The present case tells us that severe chronic hyponatremia must be managed with extreme care especially in patients with chronic debilitating illness and that relowering serum Na is a treatment of choice when CPM is suggested.

## Figures and Tables

**Figure 1:**
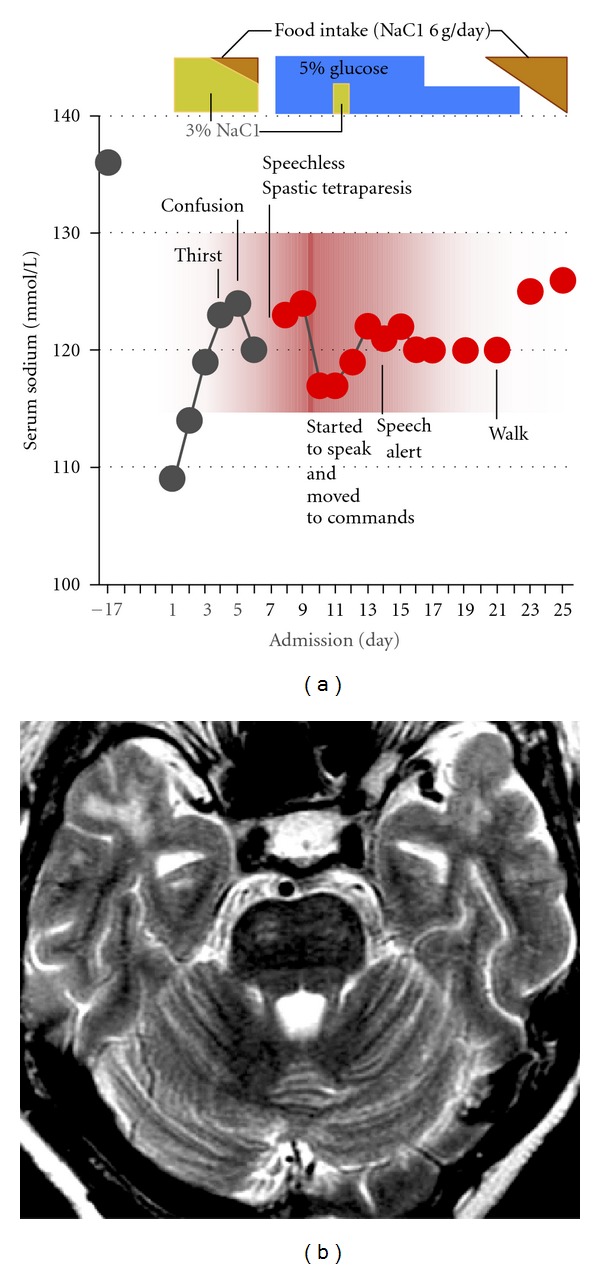
(a) Schematic presentation of the clinical course of the patient. (b) Axial, T2-weighted MR image of the pons. Three linear hyperintense lesions are observed in the center of the pons.
